# Non-coding RNA-mediated regulation of seed endosperm development

**DOI:** 10.3389/fpls.2025.1640284

**Published:** 2025-08-08

**Authors:** Xinqi Wei, Huanhuan Wang, Kaifeng Zheng, Shengcheng Han, Fanfan Zhang

**Affiliations:** ^1^ Key Laboratory of Cell Proliferation and Regulation Biology of Ministry of Education, College of Life Science, Beijing Normal University, Beijing, China; ^2^ Beijing Key Laboratory of Gene Resources and Molecular Development, College of Life Science, Beijing Normal University, Beijing, China

**Keywords:** seed, endosperm development, non-coding RNA, small RNA, long non-coding RNA

## Abstract

The endosperm, a triploid nutritive tissue in seeds, plays pivotal roles in embryo development, grain yield and quality. Recent advances highlight non-coding RNAs (ncRNAs) as central regulators of endosperm development, which integrate epigenetic, transcriptional, and post-transcriptional mechanisms. Small RNAs (sRNAs), including microRNAs and small interfering RNAs, regulate endosperm cell proliferation, starch biosynthesis, and genomic dosage response by modulating hormonal pathways, metabolic processes, and transposon silencing. Long non-coding RNAs (lncRNAs) contribute to cellularization, nutrient accumulation, and genomic imprinting via chromatin remodeling, gene expression regulation, or interactions with sRNAs. Despite growing evidence of their roles, functional characterization of ncRNAs in endosperm biology remains limited, with many regulatory mechanisms unresolved. This review synthesizes current insights into ncRNA-driven processes governing endosperm development, emphasizing the potential of ncRNAs as targets for crop improvement. Future research should prioritize functional validation of ncRNAs networks and their integration with multi-omics approaches to unlock novel strategies for precision breeding and grain trait optimization.

## 1 Introduction

The seed, a pivotal structure in plant reproduction, comprises three primary components: the seed coat, embryo, and endosperm. The endosperm, an embryonic accessory tissue, plays key roles in supporting embryo development and seed germination. Rich in starch, proteins, and lipids, it serves as the primary nutrient reservoir and supplies energy substrates for embryonic growth (Doll and Ingram, 2022). Beyond its nutritional contributions, the endosperm regulates seed morphology through physical constraints. Its expansion drives seed coat cell elongation, while its growth rate dynamically balances embryonic development to determine final seed size (Li et al., 2019; Xu and Zhang, 2023). During germination, the endosperm orchestrates hormone-mediated mobilization of stored reserves and actively remodels its cell wall structure, synergizing with the emerging radicle to breach the seed coat barrier, thereby facilitating seedling establishment (Sajeev et al., 2024; Zhao et al., 2024). In addition, as a typical triploid tissue, the endosperm exhibits unique epigenetic regulatory features conferred by its parental genomic dosage imbalance, making it a critical model for studying parental genomic interactions in plant reproductive and developmental biology (Butel and Köhler, 2024). Research on endosperm development holds significant implications: as a critical component of cereal grains, it constitutes a major global food source for humans and livestock, while also serving as a key raw material for industrial products and biofuels (Huang et al., 2021; Liu et al., 2022). Deciphering the molecular regulatory networks governing endosperm development offers precise targets for tailored improvement of grain traits, bridging fundamental plant biology and agricultural innovation.

In recent years, non-coding RNAs (ncRNAs) have emerged as a focal point in plant developmental biology due to their pivotal roles in epigenetic regulation, transcriptional control, and post-transcriptional modulation (Zhang et al., 2023c; Attallah et al., 2025). Major classes of ncRNAs include microRNAs (miRNAs), small interfering RNAs (siRNAs), long non-coding RNAs (lncRNAs), and circular RNAs (circRNAs). This review focuses on the roles of miRNAs, siRNAs, and lncRNAs in seed development, specifically within the endosperm. Distinct from protein-dependent mechanisms of coding RNAs, ncRNAs dynamically regulate spatiotemporal gene expression through intricate RNA-RNA/DNA/protein interaction networks (Ariel et al., 2015; Liu et al., 2024). Given their regulation of diverse aspects of plant growth and development, ncRNAs hold substantial implications for crop molecular breeding (Liu et al., 2025b; Yang et al., 2025). Currently, miRNAs and siRNAs have been extensively studied, whereas research on plant lncRNAs is still in its infancy. Although studies on ncRNA-mediated regulatory networks in endosperm development have gained traction, systematic reviews addressing their spatiotemporal roles and molecular mechanisms remain scarce. This review specifically examines the dynamic regulatory mechanisms of miRNAs and lncRNAs during endosperm cell proliferation and nutrient storage. Furthermore, it highlights research progress on siRNAs in controlling parental genome balance and lncRNAs in imprinting processes within the endosperm. By synthesizing cross-species evidence, we propose a multi-layered regulatory framework for ncRNAs in endosperm development to advance mechanistic insights into grain formation and inform precision molecular breeding strategies.

## 2 Seed endosperm development

The development of endosperm in angiosperms primarily exhibits three distinct patterns: nuclear-type, cellular-type, and helobial-type, with nuclear-type endosperm being the predominant developmental mode in flowering plants (Geeta, 2003). This pattern is observed in both monocot cereal crops such as rice (*Oryza sativa*), wheat (*Triticum aestivum* L.), maize (*Zea mays*), and the majority of dicot species including *Arabidopsis thaliana*, soybean (*Glycine max*), castor bean (*Ricinus communis*), and sunflower (*Helianthus annuus*).

### 2.1 Main stages of nuclear-type endosperm development

**Figure 1:**
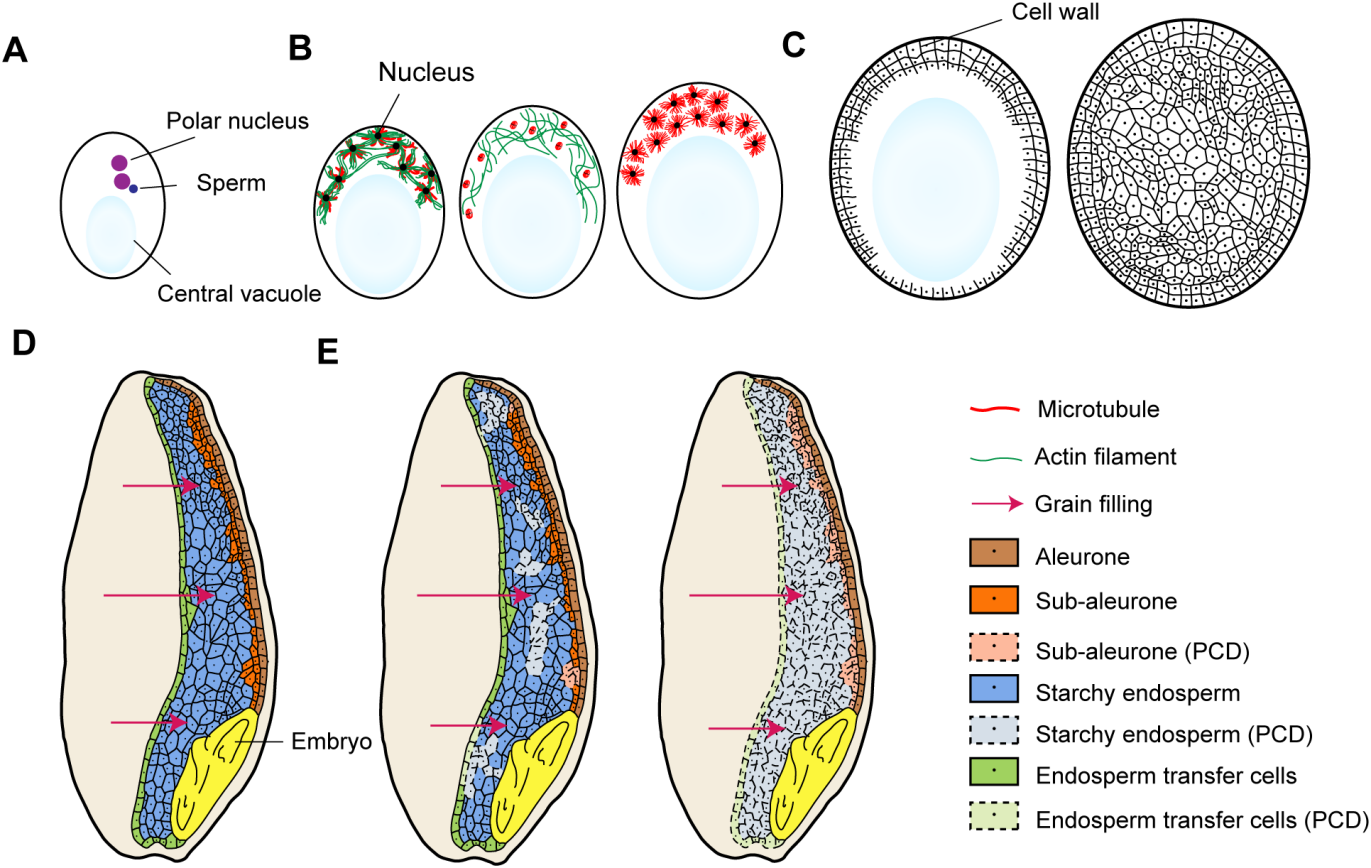
Main stages of endosperm development. **(A)** One sperm cell combines with two polar nuclei to generate triploid endosperm; **(B)** The coenocytic nuclear division stage; Microtubules and actin filaments show dynamic localizations during this stage; **(C)** The process of cellularization is typically characterized by repeated cycles of nuclear division and cell wall formation, ultimately culminating in the complete occupation of the central vacuole; **(D)** Endosperm differentiation (using wheat as an example); Wheat endosperm includes four tissue types: a aleurone layer, a sub-aleurone layer, the starch endosperm, and the endosperm transfer cells; **(E)** The PCD process along with storage product accumulation in the endosperm (using wheat as an example).

As illustrated in **Figure 1**, the development of nuclear-type endosperm involves processes such as coenocytic nuclear divisions, cellularization, endosperm differentiation, and storage product accumulation. Endosperm development initiates from double fertilization, where pollen tubes deliver two non-motile sperm cells to the female gametophyte. One sperm cell fuses with the haploid egg cell to form a diploid embryo, while the second sperm cell combines with the diploid central cell to generate triploid endosperm (Dresselhaus et al., 2016; Zhong et al., 2025) (**Figure 1A**). Post-fertilization, the central cell undergoes multiple rounds of nuclear division without cytokinesis, forming a syncytium (Boisnard-Lorig et al., 2001) (**Figure 1B**). During early syncytial development, a central vacuole emerges, displacing cytoplasm and nuclei to the peripheral region of the central cell, followed by progressive cellularization from periphery to center.

The cytoskeleton plays a pivotal role in syncytium formation and cellularization (Zhou et al., 2023), and microtubules and actin filaments show dynamic interplays (**Figure 1B**). At interphase, microtubules organize into radial arrays (asters) surrounding nuclei, maintaining inter-nuclear spacing and demarcating nuclear-cytoplasmic domains (Brown et al., 1994). At this point, F-actins also form asters and are co-located with microtubules. During nuclear division, microtubules assemble into mitotic spindles to ensure proper chromosome segregation. Along with this, F-actin asters disorganize concomitantly. This dynamic spatial regulation is critical for controlling nuclear motility and syncytium expansion. Upon cellularization, F-actin asters disappear (Ali et al., 2023), and phragmoplasts form at interfaces between adjacent microtubule asters to direct cell wall deposition (Brown and Lemmon, 2001; Bellinger et al., 2023). The cellularization process proceeds through repeated cycles of nuclear division and cell wall formation, and eventually fills the entire central vacuole (Liu et al., 2022) (**Figure 1C**).

Following cellularization, endosperm cells undergo differentiation (**Figure 1D**). In wheat, the outermost endosperm cells differentiate into the aleurone layer (Gillies et al., 2012; Liang et al., 2025), while the central cells predominantly develop into starch endosperm specialized for starch accumulation and a small amount of storage protein synthesis. A sub-aleurone layer resides as a transitional zone between the aleurone and starch endosperm (Tosi et al., 2011). Endosperm transfer cells are differentiated from the epidermal endosperm cells over nucellar projections (Olsen, 2001; Zhang et al., 2023a). Cereal species exhibit variations in endosperm cell type specification: maize endosperm additionally develops an embryo surrounding region (Dai et al., 2021), whereas endosperm transfer cells are not always recognizable in rice (Liu et al., 2022).

During mid-to-late endosperm development, all endosperm cells except the aleurone layer undergo programmed cell death (PCD), characterized by loss of plasma membrane integrity and formation of a shared cytoplasmic continuum (**Figure 1E**). This syncytial state facilitates unrestricted circulation of sugars and amino acids, driving efficient starch biosynthesis and storage protein accumulation (Liu et al., 2022). In maize, PCD initiates within the upper central endosperm and expands outward. In contrast, wheat exhibits a random PCD initiation pattern without spatial or cell-type specificity (Young and Gallie, 1999; Domínguez and Cejudo, 2014).

### 2.2 Core determinants of endosperm storage product accumulation

The accumulation of storage products in cereal grains is predominantly governed by four key biological processes: photosynthetic capacity (source strength), assimilate translocation (flow efficiency), starch biosynthesis, and endosperm cell proliferation (sink capacity) (Ma et al., 2023). Photoassimilates essential for grain filling are predominantly supplied by photosynthesis (Sanchez-Bragado et al., 2016) and transported to the developing endosperm through phloem-mediated long-distance transport for starch synthesis. Starch biosynthesis proceeds via two key biochemical steps: the conversion of sucrose into adenosine diphosphate glucose, and the subsequent conversion of this soluble precursor into insoluble starch. This metabolic cascade is tightly regulated by a suite of enzymes, including starch synthases, branching enzymes, and debranching enzymes (Emes et al., 2003). Endosperm cell proliferation directly governs starch deposition space through sink capacity modulation, wherein either reduced mitotic rates or aberrant cell cycle duration markedly compromises grain filling potential (Sahu et al., 2021). Phytohormones, notably auxin, cytokinin (CK), abscisic acid (ABA), gibberellic acid (GA), ethylene, and brassinosteroid (BR), orchestrate these four interconnected processes, serving as pivotal regulators throughout grain filling (Liu et al., 2025a).

### 2.3 Developmental divergence of endosperm in dicots vs. monocots

Endosperm development in dicotyledonous plants shares a similar developmental framework with monocot cereals, with both progressing through syncytial, cellularization, and differentiation phases. However, divergent metabolic strategies emerge during late seed maturation: dicot endosperm is progressively consumed (in part) by the embryo during seed maturation, whereas cereal endosperm persists through seed maturation with continuous starch accumulation (Olsen, 2004). Unlike other dicotyledonous plants, castor bean seeds possess a persistent endosperm, serving as a model system for studying endosperm development patterns in dicot species that retain this tissue (Xu et al., 2018).

## 3 ncRNAs in endosperm development

ncRNAs are RNA transcripts lacking protein-coding capacity (Chen and Kim, 2024), originating from intergenic regions, repetitive sequences, transposable elements, and other genomic loci (Yu et al., 2019). They comprise distinct classes including housekeeping ncRNAs and regulatory ncRNAs. This study focuses on regulatory ncRNAs, which display spatiotemporal expression specificity and regulate target genes at transcriptional or post-transcriptional levels (Erdmann et al., 2001). Based on length, ncRNAs are classified into small RNAs (sRNAs; 18–30 nucleotides), medium non-coding RNAs (31–200 nucleotides), and lncRNAs (>200 nucleotides) (Wang et al., 2017a). Recent investigations have identified functional sRNAs and lncRNAs participating in plant endosperm development.

### 3.1 sRNAs in endosperm development

Typical plant sRNAs comprise miRNAs and siRNAs, with the latter primarily categorized into heterochromatic siRNAs (hc-siRNAs) and secondary siRNAs. The biogenesis of miRNAs initiates with the transcription of endogenous *MIRNA (MIR)* genes, a process catalyzed by DNA-dependent RNA polymerase II (Pol II), which generates primary miRNAs. Due to their self-complementary nature, these primary miRNAs fold into hairpin-like structures and undergo processing mediated by DICER-LIKE (DCL) proteins to form miRNA duplexes. Subsequently, they are transported to the cytoplasm, where the mature miRNA guide strand is selectively incorporated into the RNA-induced silencing complex (RISC) to perform post-transcriptional regulatory functions (Zhao et al., 2025).

Different siRNAs exhibit distinct biogenesis pathways and functional mechanisms. hc-siRNAs are typically transcribed by the plant-specific Pol IV and mediate RNA-directed DNA methylation (RdDM), directing cytosine methylation at specific genomic loci (Onodera et al., 2005). Notably, a class of maternally derived and specifically expressed 24-nucleotide siRNAs, termed sirenRNAs (small interfering RNAs in the endosperm), has been identified in the endosperm of certain plant species (Rodrigues et al., 2013; Grover et al., 2020; Burgess et al., 2022). These sirenRNAs specifically mediate RdDM in ovule and seed tissues. Unlike canonical hc-siRNAs derived from heterochromatic transposons, sirenRNAs originate from small loci adjacent to protein-coding genes (Zhan and Meyers, 2023). Secondary siRNAs are a class of siRNAs primarily triggered by miRNAs. Among them, phased secondary siRNA (phasiRNA) precursors are typically mRNAs and lncRNAs. They are cleaved at specific positions by miRNA-loaded RISC to produce phasiRNAs, which are regularly spaced when mapped back to a precursor transcript (Liu et al., 2020). Furthermore, non-phased secondary siRNAs also exist, exemplified by epigenetically activated siRNAs (easiRNAs) enriched in pollen vegetative cells. These paternal Pol IV-associated easiRNAs contribute to rescuing hybrid seed abortion (Borges et al., 2018; Martinez et al., 2018; Satyaki and Gehring, 2019), a mechanism that will be elaborated in subsequent sections.

#### 3.1.1 miRNAs orchestrate endosperm cell proliferation

**Figure 2:**
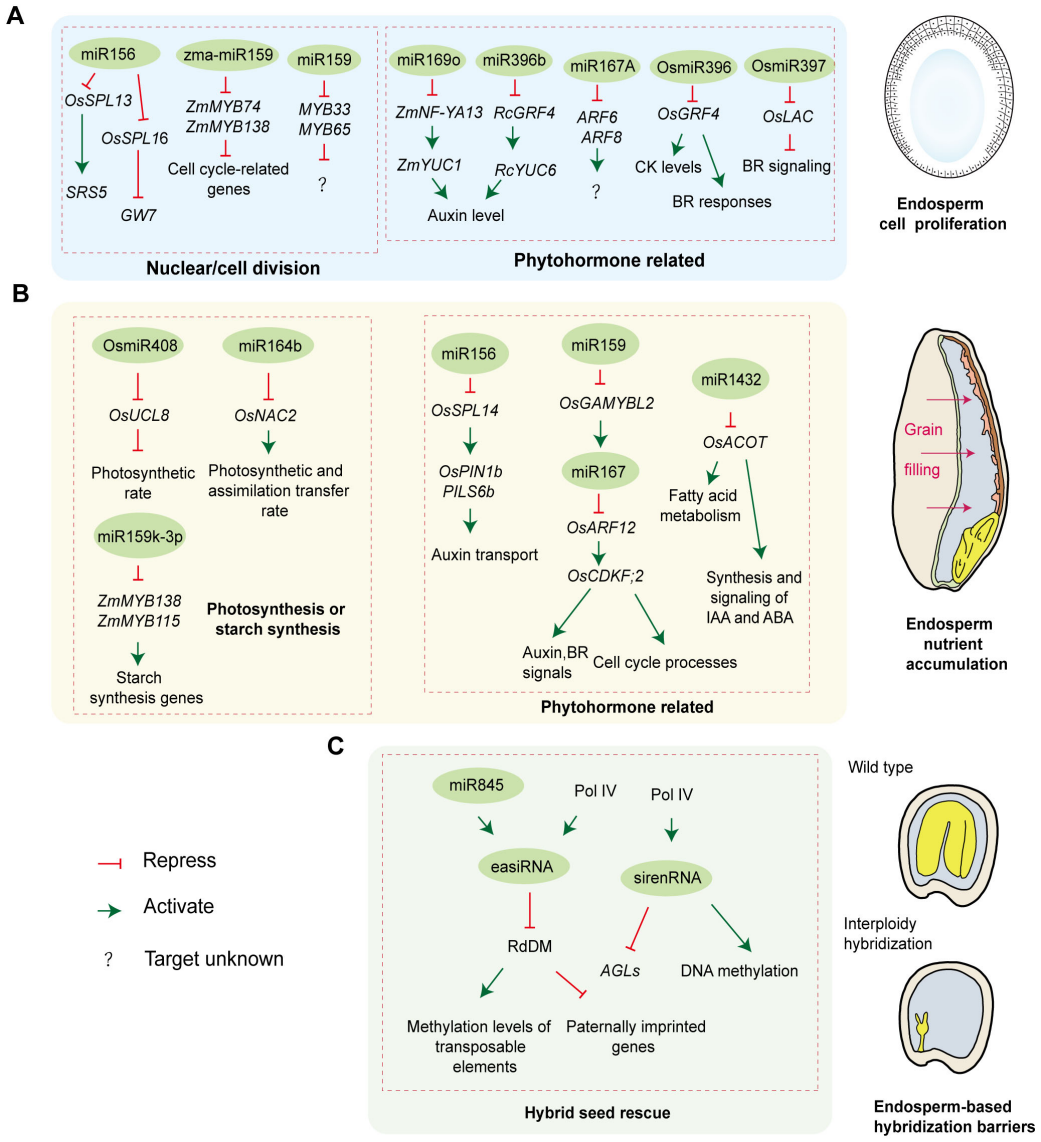
The role of sRNAs during endosperm development. **(A)** miRNAs orchestrate endosperm cell proliferation by regulating nuclear/cell division and phytohormone homeostasis; **(B)** miRNAs control endosperm nutrient accumulation by regulating photosynthesis, starch synthesis, and phytohormone pathways; **(C)** siRNAs participate in hybrid endosperm rescue by modulating epigenetic reprogramming.

The coenocytic and cellularization phases during endosperm development require interplay among networks of transcription factors, cell cycle-related genes and phytohormones. miRNAs play a central role in these complex processes by precisely regulating target genes involved in nuclear/cell division and phytohormone homeostasis (**Figure 2A**). The spatiotemporal regulatory networks of miRNAs across species provide multidimensional perspectives for deciphering the mechanisms of endosperm development.

Nuclear/cell division is a direct determinant of endosperm development and seed size. In rice, the miR156-Squamosa Promoter Binding Protein-like (SPL) module plays a central role in governing agronomic traits, including grain size, through downregulating the SPL transcription factors. OsSPL13 and OsSPL16 control grain shape through regulating cell division or expansion by altering the expression of *Small and Round Seed 5* (*SRS5*) and *Grain Weight 7* (*GW7*), respectively (Xie et al., 2006; Wang et al., 2015; Si et al., 2016). The miR159-Myeloblastosis Transcription Factor (MYB) module also acts as a regulating hub in endosperm cell division. In maize, miR159 promotes endosperm cell division and proliferation by targeting *ZmMYB74* and *ZmMYB138*, thereby alleviating their repression of cell cycle-related genes and enhancing grain size and weight (Wang et al., 2023). Notably, miR159 regulation extends beyond maternal tissues. In Arabidopsis, sperm-delivered miR159 silences maternally derived *MYB33* and *MYB65* transcripts in the central cell, lifting their repression of endosperm nuclear division. Paternal miR159 deficiency results in abnormal accumulation of *MYB33*/*MYB65* mRNAs and proteins post-fertilization, stalling endosperm nuclear division and finally causing seed abortion (Zhao et al., 2018b).

miRNAs regulate phytohormone concentration and signaling to control endosperm development. Auxin is one phytohormone attracting attention within seeds. In maize, miR169o prolongs the endosperm cell cycle by negatively regulating the transcription factor Nuclear Factor Y Subunit A13 (ZmNF-YA13), thereby suppressing its transcriptional activation of the auxin biosynthesis gene *YUCCA1 (ZmYUC1)*. This reduction in auxin level and the auxin/CK ratio ultimately increases endosperm cell numbers and expands seed size (Zhang et al., 2022). In castor bean, miR396b suppresses seed auxin levels by inhibiting the transcription factor Growth Regulating Factor 4 (RcGRF4), which in turn reduces the activation of the auxin biosynthesis gene *RcYUC6*. Paradoxically, this study revealed that lower auxin content is associated with reduced capacity for endosperm cell proliferation and expansion, and exogenous application of indole-3-acetic acid (IAA) enlarges seeds (Wang et al., 2024a). This difference may stem from the distinct developmental timing when these two miRNAs regulate auxin levels during endosperm development and/or from the fact that ncRNA-mediated regulation of auxin homeostasis in seeds is not fully conserved across species. In addition to auxin content, miRNA-mediated auxin signaling also controls endosperm development. In Arabidopsis, maternally derived miR167A is essential for endosperm development. Maternal loss of miR167A fails to repress its target genes *Auxin Response Factors 6* (*ARF6*) and *ARF8*, which function as key transcription factors for auxin response genes, finally leading to defective endosperm and seed abortion (Yao et al., 2019).

Other phytohormones also participate in regulating seed development. Rice miR397 regulates seed development through the BR pathway. Overexpression of miR397 downregulates its target gene *Laccase* (*OsLAC*), thereby promoting BR signaling, leading to increases in grain size and panicle branching (Zhang et al., 2013). In contrast to miR396’s auxin-mediated regulation of seed size in castor bean, rice studies demonstrate that the miR396-GRF4 network controls grain size by activating BR responses or elevating CK levels (Che et al., 2015; Duan et al., 2015; Sun et al., 2016). These findings highlight species-specific differences in miR396 function. They also point to miR396’s broader role in modulating multiple phytohormone pathways and their crosstalk during seed development.

#### 3.1.2 miRNAs regulate endosperm nutrient accumulation

Grain filling is the key stage of grain development determining final yield and quality. miRNAs are important regulators of grain filling (**Figure 2B**). First, miRNAs affect seed development by regulating photosynthesis or starch synthesis. In rice, overexpressing miR408 positively regulates grain yield by increasing seed number and weight. miR408 inhibits its target *Uclacyanin 8* (*OsUCL8*), which enhances photosynthetic rate by optimizing copper allocation, thus providing sufficient carbohydrates for seed development (Zhang et al., 2017). miR164b directly targets *NAM, ATAF1/2, CUC2 Transcription Factor 2* (*OsNAC2*), and plants overexpressing an *OsNAC2* mutant resistant to miR164b cleavage exhibited improved grain number and yield. Further research revealed that these transgenic rice plants have larger flag leaves and more vascular bundles, potentially leading to higher photosynthetic and assimilate transfer rates, ultimately enhancing grain yield (Jiang et al., 2018). In maize, miRNAs show temporal expression during endosperm development, preferentially repressing transcription factors related to starch synthesis genes. Specifically, miR159k-3p targeted *ZmMYB138* and *ZmMYB115*, which regulate the transcriptional activities of key starch synthesis genes (*Waxy* and *Amylose extender 1*), thereby influencing starch accumulation in the endosperm (Hu et al., 2021).

Moreover, the miRNA-mediated coordination of phytohormones and metabolic processes is essential for seed endosperm development. In rice, miR156-OsSPL14 orchestrates tiller number and seed weight through regulating the expression of *PIN-FORMED 1b* (*OsPIN1b*) and *PIN-LIKE6b* (*PILS6b*) to fine-tune auxin transport dynamics (Jiao et al., 2010; Li et al., 2022). During rice endosperm filling, suppression of miR167 expression significantly increases grain weight and yield by enhancing the grain filling rate. Mechanistically, miR167 targeted *OsARF12*, which directly binds to the promoter of *Cyclin-dependent Kinase F;2* (*OsCDKF;2*) to activate its expression. *OsCDKF;2* is involved in auxin and BR signaling and cell cycle processes, and its activation increases grain filling and size. Notably, the expression of miR167 is regulated by miR159, a positive regulator of grain size, which modulates miR167 expression through *OsGAMYBL2* (a negative regulator of GA biosynthesis) (Zhao et al., 2023). Additionally, inhibition of miR1432 enhances the grain filling rate by reducing cleavage of its target gene rice *Acyl-CoA thioesterase* (*OsACOT*) mRNA, thereby increasing *OsACOT* expression. OsACOT participates in medium-chain fatty acid biosynthesis, and its altered expression affects fatty acid metabolism as well as the synthesis and signaling of hormones such as IAA and ABA (Zhao et al., 2018a).

In recent years, predictive studies based on small RNA sequencing have provided new perspectives on miRNA regulation in endosperm development. Multiple studies have revealed numerous novel miRNAs in various crops through high-throughput sequencing. The target genes of these miRNAs are predominantly enriched in transcription factors and key metabolic enzymes, with their regulatory networks involving processes such as phytohormone homeostasis, starch synthesis, galactomannan metabolism, seed dormancy, and storage product metabolism. For instance, miRNAs in maize (Huang et al., 2016) and foxtail millet (*Setaria italica* (L.) P. Beauv.) (Li et al., 2024) target starch synthases and transcription factors to influence starch metabolism; miRNAs in Tartary buckwheat (*Fagopyrum tataricum*) (Li et al., 2021) and *Brassica napus* (Huang et al., 2013) participate in seed development and hormone responses; novel miRNAs regulating non-starch galactomannan biosynthesis have been identified in clusterbean (*Cyamopsis tetragonoloba*) (Tyagi et al., 2018); studies in rice (Panigrahi et al., 2021; Park et al., 2024) and wheat (Meng et al., 2013) have associated miRNAs with seed dormancy and grain filling processes; comparative analyses in rice have revealed differential miRNA expression patterns associated with phytohormone homeostasis and starch accumulation between superior and inferior spikelets, which exhibit distinct grain-filling rates and grain weight (Peng et al., 2011, 2014). However, most current researches are preliminary, primarily relying on differential expression analysis and bioinformatics predictions. Experimental validation through genetic transformation or functional studies remains limited, leaving the hierarchical regulatory mechanisms orchestrated by miRNAs largely unexplored.

#### 3.1.3 The role of siRNAs in endosperm-based hybridization barriers

The frequent occurrence of hybrid seed abortion following cross-species hybridization poses a persistent challenge in plant breeding, hindering the introduction of advantageous characteristics. This postzygotic hybridization barrier primarily occurs in the endosperm. siRNAs play a critical role in hybrid endosperm rescue, regulating genomic dosage responses, transposon silencing, and epigenetic reprogramming to maintain parental genome balance and determine seed viability (Ng et al., 2012; Bente and Köhler, 2024) (**Figure 2C**).

In Arabidopsis, when paternal genome dosage is doubled during interploidy crosses, excessive easiRNA accumulation interferes with the RdDM pathway, leading to a significant decrease in methylation levels of transposable elements and abnormal activation of paternally expressed imprinted genes, ultimately causing endosperm developmental defects and triploid seed abortion (Martinez et al., 2018; Satyaki and Gehring, 2019). Specifically, paternal easiRNAs are triggered by miR845, and their biogenesis depends on RNA polymerase IV activity. miR845 activates the synthesis of 21–22 nt easiRNAs in a dose-dependent manner by targeting the reverse transcription primer binding site of LTR-retrotransposons in Arabidopsis pollen (Borges et al., 2018). Remarkably, paternal Pol IV deficiency reduces easiRNA production, restoring transposable element methylation and seed viability (Borges et al., 2018; Martinez et al., 2018).

Beyond paternal mechanisms, maternal siRNAs also regulate endosperm development by suppressing parental genome conflicts through targeting the methylation network. In *Capsella* hybrid endosperm, dose-sensitive maternal sirenRNAs repress the expression of several AGAMOUS-like MADS-box transcription factor genes *(AGLs)* that target hypomethylated regions and regulates endosperm development. Insufficient sirenRNA dosage causes increased *AGL* expression and a loss of DNA methylation, triggering seed abortion in response to paternal-excess interspecies and interploidy hybridizations (Dziasek et al., 2024). A similar regulatory mechanism mediated by maternal siRNAs occurs in Arabidopsis seeds (Lu et al., 2012). Interestingly, like paternally derived siRNAs, the production of these maternal siRNAs also requires Pol IV activity. However, unlike paternal loss of Pol IV, maternal lack of Pol IV activity causes defects in chromatin condensation, DNA methylation, and hybrid seed formation (Dziasek et al., 2024). These findings suggest that maternally and paternally derived Pol IV-dependent siRNAs exert divergent regulatory roles during hybrid endosperm development.

Based on the above studies, siRNAs constitute the core molecular mechanism of endosperm hybridization barriers through dose-dependent transposable element silencing, dynamic methylation regulation, and imprinted gene expression balance. Paternally derived siRNAs and maternally derived siRNAs maintain parental genome-epigenetic homeostasis by antagonizing or enhancing the RdDM pathway, respectively. These findings may illuminate strategies for polyploid crop breeding and breaking reproductive isolation.

In summary, sRNAs orchestrate endosperm development through modular regulatory networks integrating hormone homeostasis, cell cycle progression, biomass metabolism and epigenetic elements. Future research combining multi-omics technologies and functional experiments to precisely identify and edit these networks will offer novel strategies for high-yield, high-quality breeding.

### 3.2 lncRNAs in endosperm development

In addition to the highly conserved RNA polymerases I/II/III in eukaryotes that mediate the biosynthesis of lncRNAs, plants possess evolutionarily specialized Pol IV and Pol V, which generate distinct classes of lncRNAs critical for epigenetic silencing of transposable elements (Wierzbicki et al., 2021). From a genomic structural perspective, plant lncRNAs have been systematically classified into major categories, including promoter upstream transcripts (PROMPTs), enhancer RNAs (eRNAs), long intergenic non-coding RNAs (lincRNAs), and natural antisense transcripts (NATs) (Wu et al., 2017). Functionally, they are usually categorized as *cis*-regulatory (targeting neighboring genes) or *trans*-regulatory (modulating distal genes). Mechanistically, *cis*-acting lncRNAs directly regulate chromatin architecture or transcriptional activity of adjacent genes, while *trans*-acting lncRNAs recruit chromatin-modifying complexes, interact with transcription factors, or serve as signaling hubs, decoys, guides, and scaffolds to fine-tune gene expression at epigenetic, transcriptional, or post-transcriptional levels (Pinky et al., 2023). Interestingly, some lncRNAs possess both cis- and trans-regulatory roles (Ye et al., 2022; Desideri et al., 2024), highlighting the complexity of lncRNA regulatory mechanisms.

Over the past decades, numerous lncRNAs have been identified in plant transcriptomes, yet only a fraction have been functionally characterized. lncRNAs exhibit significant regulatory potential in seed endosperm development, though no comprehensive reviews currently address this topic. Elucidating the roles of lncRNAs in endosperm cell proliferation, nutrient storage, and developmental timing could provide novel genetic resources for crop improvement.

#### 3.2.1 lncRNAs regulate endosperm cellularization and starch biosynthesis

**Figure 3:**
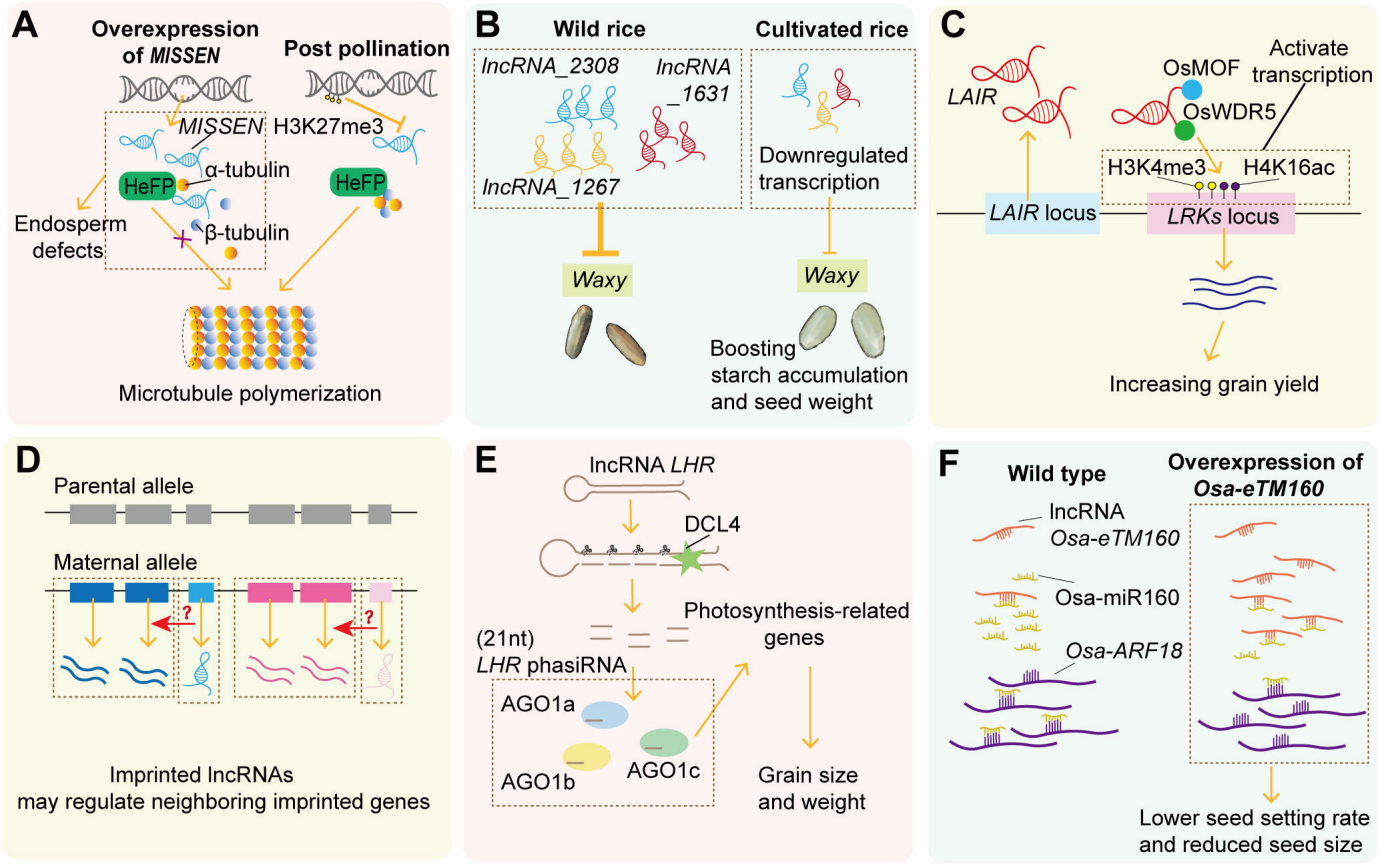
The function of lncRNAs during seed development. **(A)** The lncRNA *MISSEN* suppresses endosperm development by competing with HeFP for microtubule binding, while post-pollination H3K27me3 progressively silences *MISSEN* expression. **(B)** Three lncRNAs are downregulated during rice domestication and this directional selection on lncRNAs influences the expressions of a starch synthase-coding gene, *Waxy*. **(C)** The lncRNA *LAIR* regulates grain yield by binding histone modification proteins OsMOF and OsWDR5 to induce epigenetic changes and activating its neighboring gene *LRK1*. **(D)** Maternally imprinted lncRNAs cluster with imprinted protein-coding genes, suggesting functional roles within imprinting clusters in endosperm. **(E)** The lncRNA *LHR* forms a hairpin structure to generate phasiRNAs via DCL4 and a miRNA-like pathway, essential for seed development by targeting photosynthesis-related genes. **(F)** The lncRNA *osa-eTM160* acts as an osa-miR160 target mimic to release *osa-ARF18*, thereby regulating grain size and seed-setting rate.

Endosperm development governs seed morphogenesis and nutrient accumulation efficiency through tightly orchestrated processes such as syncytium formation, cellularization, differentiation, and nutrient accumulation. Recent studies reveal that lncRNAs dynamically regulate key biological processes in endosperm development—such as cell cycle progression, cytoskeleton remodeling, and starch biosynthesis—forming intricate molecular regulatory networks (**Figure 3**).

A seminal example is the maternal lncRNA *Mis-Shapen Endosperm* (*MISSEN*) discovered in rice (**Figure 3A**). Through a genome-wide screen of lncRNAs during rice sexual reproduction, a T-DNA insertion mutant of *MISSEN* was identified with low fertility and mis-shapen seed phenotype (Zhang et al., 2014). Further functional analysis revealed that the T-DNA insertion was an activating mutation with higher *MISSEN* levels, and it produced a similar phenotype to *MISSEN* overexpressing plants, suggesting that *MISSEN* negatively regulates endosperm development (Zhou et al., 2021). Mechanistically, *MISSEN* binds specifically to the helicase family protein HeFP, competitively disrupting HeFP-microtubule interactions. This leads to aberrant microtubule polymerization, nuclear division defects, and delayed endosperm cellularization. Notably, *MISSEN* knockdown lines exhibit accelerated cellularization and enlarged grain size, highlighting the *MISSEN*-HeFP pathway as a potential target for improving grain yield. Moreover, the author also found that epigenetic modifications H3K27me3 progressively suppress *MISSEN* expression post-pollination, unveiling a dosage-dependent regulatory logic in which maternal lncRNAs precisely control endosperm developmental timing. This study provides a paradigm for functional characterization of lncRNAs during seed and endosperm development.

In metabolic pathway regulation, lncRNAs play particularly critical roles in controlling endosperm starch biosynthesis. During seed development, stage-specific lncRNAs are significantly enriched in carbohydrate metabolism, starch synthesis, and transport-related pathways (Zhang et al., 2023d). In line with this, a genome-wide analysis of early panicles in cultivated rice and wild rice revealed that most differentially expressed lncRNAs are downregulated during domestication, with this directional selection primarily influencing the expression of energy metabolism genes related to carbohydrate transport and accumulation (Zheng et al., 2019) (**Figure 3B**). Transgenic overexpression of these three wild rice lncRNA alleles (*LncRNA_2308*, *LncRNA_1267*, and *LncRNA_1631*) in cultivated rice resulted in higher grain chalkiness and lower grain weight compared to controls. Notably, expression of *Waxy*—encoding granule-bound starch synthase, a key regulator of grain starch content—was significantly reduced in lncRNA-transgenic plants. This study demonstrates that domestication-driven selection for targeting lncRNA suppression represents a critical mechanism for enhancing starch accumulation and seed weight. Consistent with the *MISSEN* regulatory framework, these lncRNAs likely function as inhibitory regulators during seed development. Investigating the molecular mechanisms underlying lncRNA suppression may therefore uncover novel genes functionally linked to endosperm development.

Endosperm starch contains amylose and amylopectin, with high-amylose starch considered resistant (“healthy”) starch beneficial for preventing diet-related diseases like obesity. In bread wheat endosperm, lncRNA *TCONS_00130663* shows strong correlation with *SBEIIb* (encoding starch branching enzyme) and *LYPL* (encoding lysophospholipase), indicating that lncRNAs may regulate resistant starch biosynthesis through unknown mechanisms (Madhawan et al., 2020). Storage proteins, another key endosperm component determining grain quality, also show co-expression patterns with specific lncRNAs, which may regulate enzymes involved in storage protein biosynthesis (Zhang et al., 2023d).

Several lncRNAs regulate seed yield traits, though endosperm-specific roles are not always confirmed. For example, the lncRNA *leucine-rich repeat receptor kinase antisense intergenic RNA* (*LAIR*) is a positive regulator of seed production and its overexpression increases yield-related traits including grain number and panicle number (**Figure 3C**). Further, it was revealed that *LAIR* induces epigenetic changes through specifically binding histone modification proteins like Males Absent on the First (OsMOF) and WD Repeat Domain 5 (OsWDR5), and then activates the expression of its neighboring yield-associated gene *Leucine-rich Repeat Receptor Kinase* (*LRK1*) (Wang et al., 2018). A recent study found that *LAIR* has nine alternative splicing isoforms and different compositions of *LAIR* isoforms could fine-tune *LRK1* expression and seed yield under different abiotic stress (Wang et al., 2024b).

Several studies using genome-wide analysis to identify lncRNAs candidates regulating seed development have been carried out in rice, maize, barley (*Hordeum vulgare* L.), castor bean, rapeseed (*Brassica napus*), and *Jatropha curcus*, etc (Shen et al., 2018; Wang et al., 2019; Yan et al., 2020; Zhao et al., 2020; Zou et al., 2020; Pinky et al., 2023; Zheng et al., 2023). They have identified differentially expressed lncRNAs participating in starch/sucrose metabolism, gene expression regulation, and cell growth. While these provide comprehensive catalogs of lncRNAs potentially regulating endosperm development, extensive functional validation is required to elucidate specific molecular mechanisms.

#### 3.2.2 The role of lncRNAs in endosperm imprinting

Parental epigenetic asymmetry leads to monoallelic gene expression, a phenomenon termed genomic imprinting. In plants, imprinting primarily manifests in the endosperm, reflecting divergent epigenetic compositions between parental gametes (Muthusamy et al., 2024). Several lncRNAs exhibit imprinting effects, manifesting allelic expression divergence in plants. In hybrid endosperm of castor bean, most allelic lncRNAs displayed dosage insensitivity while conforming to the expected 2:1 maternal-to-paternal expression ratio. However, a small subset of allelic lncRNAs significantly deviated from the expected ratio, predominantly at genomic loci where substantial parental expression divergence was detected. This differential expression of allelic lncRNAs partially reflects parent-of-origin effects or genomic imprinting. Maternally imprinted lncRNAs were consistently clustered with maternally imprinted protein-coding genes, with some demonstrating strong positive correlation with adjacent imprinted protein-coding genes (**Figure 3D**). These findings suggest that imprinted lncRNAs may function within imprinting clusters.

Consistent with this, genome-wide analysis in rice endosperm identified 16 intergenic imprinted lncRNAs linked to imprinted genes. They were located near imprinted genes, or were transcribed oppositely to adjacent imprinted alleles, implying *cis*-regulation of neighboring imprinted genes (Yuan et al., 2017). Studies in maize have revealed more complex regulatory patterns: four maternally imprinted intronic lncRNAs are transcribed from paternally expressed genes. For instance, the *GRMZM2G477503* locus produces a maternally expressed lncRNA (*ZmMNC18*) from its third intron, while its exonic regions exhibit paternal-specific expression. Zhang et al. hypothesize that these maternal lncRNAs may recruit silencing complexes to repress maternal alleles, thereby establishing paternal dominance in expression of these genes (Zhang et al., 2011).

Previously mentioned *MISSEN* is also maternally imprinted. CRISPR-Cas9-edited *MISSEN* transcripts were expressed in endosperm exclusively when mutant plants served as female parents, confirming maternal-specific expression (Zhou et al., 2021). Collectively, these studies suggest that imprinted lncRNAs may play specific roles in endosperm development.

Despite progress, research on imprinted lncRNAs remains in its infancy, with studies limited to few species. Mechanistic interactions between imprinted lncRNAs and genes require deeper validation, and connections to seed agronomic traits are underexplored.

#### 3.2.3 lncRNA and sRNA crosstalk in seed endosperm

lncRNAs can interact with sRNAs as precursors or endogenous miRNA target mimics (eTMs). A genome-wide identification in maize revealed >90% of lncRNAs are predicted sRNA precursors (Li et al., 2014). While proportions vary across species/studies (Liu et al., 2012; Palos et al., 2023), experimental evidence confirms that lncRNAs function as sRNA precursors during seed development. For example, rice lncRNA *Long Hairpin-structure containing noncoding RNA* (*LHR*) forms a hairpin structure serving as a phasiRNA precursor (Huang et al., 2019) (**Figure 3E**). Intriguingly, *LHR*-derived phasiRNA biogenesis depends on DCL4—but not RDR2/6—via a miRNA-like pathway. A T-DNA insertion mutant *lhr*, which blocks hairpin structure formation, causes multiple developmental defects including reduced rice grain size and weight. Degradome analysis suggests *LHR*-phasiRNAs target thylakoid-related genes to regulate photosynthesis, highlighting the importance of *LHR*-mediated phasiRNA production in seed development.

Another rice lncRNA, *osa-eTM160*, counteracts the inhibitory effects of osa-miR160 on *osa-ARF18* transcripts through target mimicry, thereby influencing grain size (Wu et al., 2013; Wang et al., 2017b) (**Figure 3F**). Transgenic plants overexpressing *osa-eTM160* exhibited reduced osa-miR160 levels, while plants expressing a mutant *osa-eTM160* (insufficient miRNA pairing) exhibited no such decrease. Moreover, *osa-eTM160* and *osa-ARF18* share similar expression profiles, and their overexpression similarly reduces seed setting rate and seed size. This study highlights how lncRNAs act as stage-specific modulators to fine-tune sRNA-mediated gene regulation during restricted developmental windows of seeds.

Studies in other species have also initially revealed the interaction between lncRNAs and miRNAs in endosperm. For example, in wheat grains, lncRNA *TCONS_00130663* interacts with miR1128, potentially regulating *LYPL* for lipid biosynthesis (Madhawan et al., 2020), implying an lncRNA-miRNA-mRNA regulatory axis during endosperm development. In maize seeds, a transcriptomic analysis revealed that four endosperm-specific expressed lncRNAs (*lncRNA_71309*, *lncRNA_02785*, *lncRNA_86055*, and *lncRNA_58195*) may act as eTMs in lncRNA-miRNA-mRNA co-expression networks enriched for carbon fixation, BR biosynthesis, and lipid metabolism (Zhu et al., 2017).

Taken together, current research has unveiled lncRNAs’ cellular regulation, metabolic intervention, and miRNA interaction mechanisms in endosperm development, providing multidimensional insights into seed development networks. Nevertheless, despite advancing omics screening, cross-species evolutionary conservation analyses and dynamic expression landscapes across entire grain developmental stages of lncRNAs still lack. Moreover, mechanistic studies on specific lncRNAs remain inadequate, constituting key bottlenecks for functional characterization and breeding applications.

## 4 Conclusions and perspectives

Endosperm development serves as a key mechanism in plants, crucially influencing both seed yield and quality characteristics. Extensive studies have revealed that ncRNAs act as central regulatory components in seed endosperm development by integrating cellular, hormonal, and epigenetic regulatory factors. In light of the present findings, future breeding efforts on manipulating ncRNAs in seeds offer the following advantages. Firstly, plant genomes transcribe significantly more ncRNAs than protein-coding genes. Thus, genetic manipulation of ncRNAs holds great promise for providing a wealth of untapped genetic resources for precision breeding in crops. Secondly, ncRNAs are involved in almost every aspect of plant growth and development and regulate key agronomic traits such as grain yield, fertility, stress response, disease resistance, and nutrient utilization. Combining non-coding genes and coding genes that govern different agricultural traits in molecular design breeding will facilitate the development of superior varieties with multiple desirable traits. Thirdly, ncRNA expression is generally tissue-specific and diverse. By altering ncRNA expression levels, it is possible to finely adjust the expression of target genes without significantly disrupting protein function, thereby reducing the negative effects associated with modifying critical protein-coding genes. This approach is conducive to achieving trade-offs between different target traits, such as grain yield and quality, plant yield and disease resistance, etc.

Over the past few decades, although functional studies of sRNAs in seeds have advanced significantly, a large number of sRNAs remain uncharacterized. Current research on siRNA regulation of seed development is relatively limited, and the targets of some reported miRNAs and siRNAs remain unknown. Furthermore, many studies have only found that miRNAs regulate seed size, but the specific stages and processes of their involvement in seed development are not well defined. Integrating single-cell sequencing and multi-omics analyses will be critical to identifying sRNAs that play essential roles during specific stages of endosperm development.

In contrast to sRNAs, the functions of the vast majority of lncRNAs in seed remain largely unknown, with functional research lagging far behind bioinformatics screening. At present, the research on plant lncRNAs is facing a series of challenges. Firstly, the current nomenclature for lncRNAs in plants is highly inconsistent, severely hindering information exchange and data sharing. There is an urgent need to establish a unified nomenclature and definitions based on nucleic acid sequences and genomic locations. Secondly, the regulatory mechanisms of lncRNAs are diverse and highly variable, currently with each being distinct, posing significant challenges to research. Thirdly, the low evolutionary conservation of lncRNAs renders traditional functional studies—conducted separately across species—prohibitively costly. Nevertheless, cross-species and cross-organ analyses of lncRNAs remain feasible in mammals (Sarropoulos et al., 2019), suggesting latent potential for similar approaches in plants. To bridge this gap, plant research must leverage artificial intelligence (AI) and machine learning to decode the developmental dynamics and conservation patterns of lncRNAs. Ultimately, mining core conserved lncRNAs governing key agricultural traits, combined with AI-assisted molecular design breeding and phenomics, will establish a foundational framework for cross-species lncRNA utilization. Emerging regulatory layers revealed by recent studies encompass lncRNA interactions with phase separation machinery, RNA modifications, and 3D genome architecture (Guo et al., 2021; Luo et al., 2021; Zhang et al., 2023b; Hu et al., 2024; Lei et al., 2025; Tian et al., 2025). Targeted screening of interactions between proteins and lncRNAs involving these layers (phase separation, RNA modification, or 3D genome architecture) may help identify key regulatory lncRNAs during endosperm development and provide novel genetic resources for crop improvement.
